# Cognitive changes and neural correlates after oral rehabilitation procedures in older adults: a protocol for an interventional study

**DOI:** 10.1186/s12903-021-01654-5

**Published:** 2021-06-09

**Authors:** Linn Hedberg, Urban Ekman, Love Engström Nordin, Jan-Ivan Smedberg, Pia Skott, Åke Seiger, Gunilla Sandborgh-Englund, Eric Westman, Abhishek Kumar, Mats Trulsson

**Affiliations:** 1 Folktandvården Eastmaninstitutet, Stockholm, Sweden; 2 Division of Oral Diagnostics and Rehabilitation, Department of Dental Medicine, Karolinska Institutet, Alfred Nobels Allé 8, Box 4064, 141 04 Huddinge, Sweden; 3 Division of Clinical Geriatrics, Department of Neurobiology, Care Sciences and Society (NVS), Karolinska Institutet, Huddinge, Sweden; 4 Department of Diagnostic Medical Physics, Karolinska University Hospital, Solna, Stockholm, Sweden; 5 Academic Centre for Geriatric Dentistry, Stockholm, Sweden

**Keywords:** Chewing performance, Magnetic resonance imaging, Episodic memory, Executive functions, Visuospatial functions, Logical thinking

## Abstract

**Background:**

Epidemiological studies show an association between masticatory function and cognitive impairment. This has further strengthened the notion that tooth loss and impaired masticatory function may be risk factors for dementia and cognitive decline. Animal experiments have indicated a causal relationship and several possible mechanisms have been discussed. This evidence is, however, lacking in humans. Therefore, in the current interventional study, we aim to investigate the effect of rehabilitation of masticatory function on cognition in older adults.

**Methods:**

Eighty patients indicated for prosthodontic rehabilitation will be randomly assigned to an experimental or a control group. Participants will conduct neuropsychological assessments, masticatory performance tests, saliva tests, optional magnetic resonance imaging, and answer questionnaires on oral health impact profiles and hospital anxiety and depression scale before, 3 months, and 1 year after oral rehabilitation. The difference between the two groups is that the control group will be tested an additional time, (at an interval of about 3 months) before the onset of the oral rehabilitation procedure. The primary outcome is a change in measures of episodic memory performance.

**Discussion:**

Although tooth loss and masticatory function are widespread in older people, it is still an underexplored modifiable risk factor potentially contributing to the development of cognitive impairment. If rehabilitation of masticatory function shows positive effects on the neurocognitive function, this will have great implications on future health care for patients with impaired masticatory status. The present project may provide a new avenue for the prevention of cognitive decline in older individuals.

*Trial registration*: The protocol for the study was retrospectively registered in ClinicalTrials.gov Identifier: NCT04458207, dated 02-07-2020.

## Background

Dementia is a general term that encompasses several neurodegenerative diseases that affect approximately 7% of the population older than 65 years [1], and about 30% older than 80 years [2, 3]. The World Health Organization defines dementia as a “syndrome in which there is deterioration in memory, thinking, behavior, and the ability to perform everyday activities”. Studies have suggested a rapid rise in the incidence of dementia in the aging population [4, 5]. Since dementia is a chronic and progressive syndrome, its prevalence will also increase with the rise of the aging population. Further, with the increase in life expectancy, the global burden for the health care of individuals with dementia is expected to increase exponentially. Therefore, the steep rise in dementia is presenting a "significant and urgent challenge" to health care services, and interventions are needed to prevent and counter these challenges [6].

Current research has emphasized the concept of a “brain-stomatognathic axis” in relation to geriatric healthcare. Accordingly, this axis is the complex communication network between the cortical and subcortical regions of the brain and the stomatognathic/masticatory system [7–9]. Oral conditions such as loss of teeth [10, 11], chronic inflammatory disease such as periodontitis [12], and chewing difficulties [13] are commonly associated with the risk of neurodegenerative diseases [14–18]. Evidence from animal studies has further strengthened the associations and has suggested a causal relationship.

Studies on aged rats and mice conclude that a disturbance of normal mastication accelerates the age-dependent impairment of learning abilities and memory, combined with significant degenerative changes in the hippocampus [19, 20]. Specifically, the linkage between mastication and brain function in rodents has been studied by either altering the diet (i.e., hard food vs powder or liquid diet) [21], alteration of occlusion [22, 23], or deliberate extraction of teeth [20, 24–26]. Studies have reported impaired memory and learning ability with increased expression of brain-derived neurotrophic factor (BDNF) and the decreased number of pyramidal neurons in the hippocampus in mice fed on liquid diets [27]. Studies have also reported that cell proliferation in the hippocampus is characteristically inhibited by soft diet feeding in rats [28]. Further, it was shown that loss of masticatory function by deliberate extraction of maxillary molars in young growing mice resulted in chronic stress and malnutrition. These mice developed impaired hippocampal-dependent learning ability, hyperactivation, and lateralized rotation behavior commonly associated with dysfunction of the dopaminergic system [24]. The dopaminergic system plays a vital role in attention and recognition memory in the prefrontal cortex and the hippocampus [24].

Human epidemiological studies have consistently suggested correlations/associations between masticatory impairment and a low number of teeth with the deterioration of cognition and memory [13, 15, 29–32]. Further, magnetic resonance imaging (MRI) studies have confirmed the association between chewing and increased activation of memory centers of the brain [33, 34]. It is also suggested that successful aging, i.e., maintaining high cognitive capacity, might partly be affected by preserving the natural teeth [35]. However, it is also debated whether the associations between the indicators of poor oral health (dentition status, periodontal disease) and cognitive health could be bidirectional. Patients with impaired cognitive function may be more likely to have poorer oral hygiene, resulting in periodontal disease and tooth loss, due to their cognitive problems. On the one hand, several studies investigating oral health (i.e., periodontitis) as an independent risk factor for dementia have had a cross-sectional study design and thereby problems with temporality and reverse causality. Then again, studies do show significant correlations and associations between chewing and cognitive functions. Thus “cause-effect” relationships have yet to be established in humans. Therefore, the question whether the detrimental effect of loss of masticatory function on cognition is reversible through oral rehabilitation remains to be investigated. In the current project, we propose an interventional study to investigate the relationship between mastication and cognition in humans. The specific aim of the study is to investigate the effect of rehabilitation of masticatory function on neurocognitive assessments in older adults.

## Methods/design

### Study location

Patient recruitment will be performed at the Swedish public dental health service, Folktandvården Stockholms Län AB, Sweden. The dental rehabilitation procedures of the recruited study participants and all the cognitive measurements; masticatory ability and masticatory function test and saliva samples will be performed/collected at the Department of Prosthetic Dentistry, Folktandvården Eastmaninstitutet, Stockholm, Sweden. The imaging data will be collected at the Stockholm University Brain Imaging Centre (SUBIC) and analyzed at the Department of Neurobiology, Care Sciences and Society (NVS), Karolinska Institutet, Sweden.

### Study participants

The study will include patients seeking fixed prosthodontic treatment either due to reduced dentition measured by Eichner’s index [36] and complaining of impaired chewing ability due to a reduced number of posterior functional units or reduced dentition in general. Efforts will be made to recruit an equal number of men and women. The criteria for inclusion (Table 1) are patients in the age range of 65–80 years, in need of prosthodontic treatment, and presenting a Mini-Mental State Examination (MMSE) score > 25 [37]. The MMSE is a screening tool that has shown to be a sensitive marker of overt dementia and will therefore help us to discriminate between normal cognitive status and individuals at risk of dementia. However, MMSE is not a cognitive test that is suitable for intervention evaluation because many patients will perform at a maximum or near-maximum level (i.e., ceiling effect), leaving no opportunity to detect improvement across time [38]. On the contrary, our outcome tests with the neuropsychological assessments (NA) are highly sensitive to change (see heading outcome parameters and measurements). Thus, we aim to exclude patients with overt dementias (with low potential of cognitive improvement after intervention), but we remain open to including patients within the cognitive continuum from normal performances to mild cognitive impairments (with higher potential of cognitive improvement by the intervention). Circumstances that could compromise the validity of the tests, such as poor Swedish language skills, reading disabilities, severely reduced hearing or vision, are also considered as a criterion for exclusion, depending on the severity of the condition. Patients without any subjective masticatory dysfunction and patients that will be rehabilitated with removable dentures are excluded. Specific criteria are used to determine eligibility for the (optional) MRI acquisitions (Table 1). The demographic details (height, weight, etc.,) along with self-reports of ongoing medications (if any) and chewing ability are collected. The participants are screened with Alcohol Use Disorders Identification Test (AUDIT) [39, 40] and the oral health-related QoL of the participants is assessed with Oral Health Impact Profile (OHIP-14) [41]. The hospital anxiety and depression (HAD) scale will be used to measure the anxiety and depression in the groups [42]. The number of remaining teeth and occluding units are collected from the dental record (intraoral photos and dental X-rays).

**Table 1:** Inclusion and exclusion criteria for study participants

Inclusion criteria	Exclusion criteria
65–80 years of age at the start of the study	Brain trauma or stroke < 6 months
Impaired chewing ability	Neurological disease (stroke, Alzheimer's disease, other dementias, Parkinson's disease)
Missing teeth as indicated by Eichner index B2-B4, C1-C4	Other intellectual disability
Indications for treatment with fixed prosthodontics, implant and/or tooth-supported (overdentures included)	Psychological disorders
Mini-Mental State Examination (MMSE) score > 25	Participants with chronic pain, depression, or sleeping disorders
	Daily analgesic medication that may affect cognitive and/or executive performance of the brain
	Poor Swedish language skills, reading disabilities
	Severely reduced hearing or vision
	*Exclusion criteria* *Magnetic resonance imaging*
	Claustrophobia
	Difficulties in lying down in a supine position for about an hour, or any other difficulties related to the MRI head coil
	Participants with mental or medical implants in the body contraindicating MRI scan

### Sample size

No studies are currently available that directly compare rehabilitation of chewing function with a control condition on neurocognitive measures. We, therefore, designed our RCT as a superiority trial with enough statistical power to detect a difference in outcome between treatments (if present) with a medium effect size (partial eta square, eta2). For the longitudinal analysis that assesses rehabilitation differences, we expect an effect size (partial eta square, eta^2^) on the NA of at least 0.06. With α set to 0.05 and power at 0.80, a sample of 30 participants in each group is required. The power calculation of the repeated measures analysis is estimated with within-participant factors, which also controls for the between-participant variance. However, due to the moderate rate of dropouts (20–25%) observed across different ongoing studies, optional non-participation of MRI screening, and technical difficulties (e.g., movement artifacts) during brain imaging, we estimate that inclusion of 40 participants in each group is required to meet the demands for statistical power. However, only the first 20 participants willing and eligible to participate in the MRI procedures in each group will undergo brain imaging due to practical and logistical reasons. The MRI acquisitions are optional since there are more contraindications medically and patients are more reluctant to participate. By making MRI optional participants can still be included in the study when MRI acquisitions are not feasible.

### Study design

**Fig. 1:**
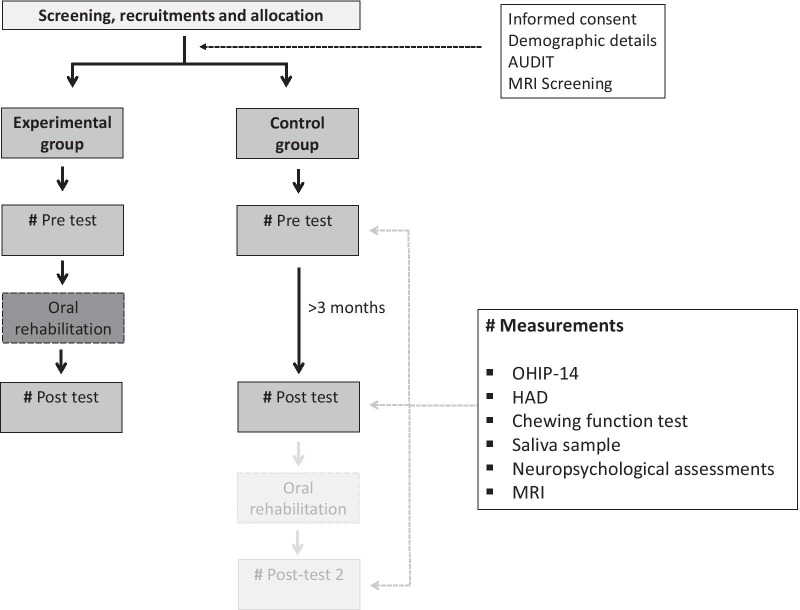
Overview of the study protocol

The study protocol and the measurements are presented in Fig. 1. As stated above, after informed consent and preliminary screening of the participants who have performed the MMSE and found eligible for inclusion in the study will also provide their basic demographic details (height, weight, etc.), self-reports of ongoing medications, along with the AUDIT and MRI screening questionnaire. The recruited participants indicated for prosthodontic rehabilitation are randomly allocated to either the experimental or the control group (see Fig. 1). A randomization list (www.randomization.com) is prepared by a nonparticipating member in the study and the randomization code is allocated over email upon request from the examiners. Further, all tests will be conducted according to the study protocol and at every test occasion, the participants will conduct saliva samples, mixing ability test, NA, questionnaires (OHIP-14, HAD) together with optional MRI acquisitions.

The oral rehabilitation of the experimental group will start immediately after the first NA (pre-test). The control group on the other hand will be pre-and post-tested with an interval of about 3 months before the onset of the prosthodontic rehabilitation procedure. The relatively short period between the pre-and post-tests for the control group is motivated by ethical considerations. It is not considered justifiable to deny prosthodontic rehabilitation to the control group any longer than 3 months. Therefore, it is likely that the time between pre-and post-test for the control group will be slightly shorter than for the experimental group. We aim to statistically control for time differences by including time between pre-and post-test as a covariate of interest in the model. Both the experimental and the control group will also perform the post-tests (see Table 2) on two occasions after the completion of the oral rehabilitation procedures (Fig. 1). The first post-test occasion is after at least 3 months and the second test is to be conducted at 1-year follow-up, approximate time needed to adapt to the new fixed prosthesis.

**Table 2:** Outcome measures

	Variable	Method/test	Measurements
*Primary outcome variables*			
1	Neuropsychological assessments	Episodic memory	BVMT-R
*Secondary outcome variables*			
2	Chewing function	Two-color chewing gum mixing test	VOH scores
3	Oral health	Oral health impact profile (OHIP) 14	OHIP score
4	Stress	Salivary test Hospital and anxiety depression scale questionnaire	Salivary Cortisol HAD score
5	Dentition status	Dental records, photos and X-rays	Eichner’s index Number of teeth Number of occluding units
6	Brain scans	Magnetic Resonance Imaging	Volume change hippocampus and/or total volume change gray matter

### Intervention: oral rehabilitation

Individual treatment options will be discussed with the patients and rehabilitation will be provided depending on the clinical situation and the patient’s financial status. The rehabilitation will include fixed prosthodontics and will be achieved by providing the latest and most effective measures in modern-day prosthodontics. Briefly, the procedures will involve a control phase involving scaling, root planning, oral hygiene instructions, etc. When needed, extractions and bone augmentation will be performed, and also temporary removable dentures will be made if required. This will be followed by the restoration of the vertical dimension with occlusal splints (if needed), tooth preparations, placement of dental implants (if needed), and finally cementation of dental crowns. The entire rehabilitation phase is estimated to take approximately 3–12 months, where implant treatment is the main factor for the longer time frames.

### Outcome parameters and measurements

Test measures are NA with good psychometric properties, i.e., good reliability (with minimum measurement errors), validity (measures the construct intended), and responsiveness (sensitivity to detect changes over time). The primary outcome is the changes in episodic memory and learning measured by the Brief Visuospatial Memory Test-Revised (BVMT-R) [43]. All secondary outcomes have been listed and summarized below in Tabel 2, two of them are corresponding changes in neuro correlates at MRI and the masticatory performance assessed by the mixing degree of the two colored gum.

*Masticatory function* The chewing function will be assessed both objectively and subjectively. The masticatory function will be evaluated using a well-established two-color chewing gum mixing test (Orophys smart chew GmbH, Bern, Switzerland) during a standardized and deliberate chewing sequence of 20 cycles in accordance with the recommended instructions [44, 45]. Accordingly, the participants will be asked to chew on the two-colored chewing gum on their preferred chewing side. The participants will be abruptly stopped by the examiner and asked to spit out the chewing gum after twenty chewing cycles. The examiner will silently count the number of chewing strokes during the tests. The chewed samples will be photographed with a phone camera (iPhone, Apple Inc.) on a specially designed stand before flattening the sample into a wafer of 1 mm thickness. The images will be imported into a photo‐editing software (Adobe Photoshop® Elements for Windows, Adobe systems incorporated, San Jose, CA, USA), both sides of the wafers will be assembled into a single image file. These files are then to be imported and analyzed in a purpose‐built freeware (ViewGum©, dHAL Software, Greece, www.dhal.com) which calculates the Variance of Hue (VoH), a parameter on the color mixing of the chewing gum.

The oral health-related QoL assessed by the participants will be evaluated with the OHIP-14 questionnaire and subject-based information on chewing ability will also be collected. These measurements will be used to assess the degree of functional impairment (pretest) and the subsequent effect of restoration of function (post-test) on masticatory function.

*Neuropsychological assessments* The NA are approximated to 90 min per test occasion and are conducted prior to the protocol by a research assistant blinded to the randomization. The neuropsychological assessments are state-of-the-art tests with good psychometric validity translated into Swedish. The aim is to use the cognitive tests that are sensitive to change and measure a broad range of cognitive domains.

The assessed cognitive domains and specific tests are:

*Episodic memory* The Ray Auditory Verbal Learning Test (RAVLT) [46], the BVMT-R [43].

*Executive functions* The Trail Making Test (TMT) trial 4 from Delis-Kaplan Executive Function System (D-KEFS) [47], the Verbal Fluency (VF) test from D-KEFS, and the Color-Word Interference Test from D-KEFS.

*Attention/working-memory* The Digit Span from Wechsler Adult Intelligence Scale (WAIS) 4th edition[48], the Digit Symbol from WAIS-IV, and the Trail Making Test (TMT) trial 1–3, and 5 from D-KEFS.

*Visuospatial functions* The Block Design from WAIS-IV.

*Logical thinking* The Matrix Reasoning from WAIS-IV.

*Brain imaging* All the Magnetic Resonance Imaging (MRI) acquisitions will be conducted at SUBIC using a Siemens Prisma 3 T scanner (Erlangen, Germany). The duration of the MRI protocol is approximately 40 min and it includes a high resolution sagittal T1-weighted MPRAGE sequence for structural changes, a T2-FLAIR sequence to exclude other types of pathologies such as tumors, and detection of white matter pathology. Further, for the resting-state functional MRI (i.e., the participants don’t perform any cognitive task but are instructed to stay awake during the scanning), an EPI blood oxygen level-dependent (BOLD) sequence will be used. A Pseudo-Continuous Arterial Spin Labeling (pCASL) sequence will be performed to measure brain perfusion. Finally, we also aim to include measures of the masseter muscle in the MRI to be able to study pre- and post-muscle volume using a PD-weighted SPACE sequence. The MRI data will be stored and analyzed within TheHiveDB which is an encrypted web-based neuroimaging database system capable of managing data for large longitudinal projects [49]. The system has the capacity to perform fully automated analysis of structural and functional imaging data using a variety of algorithms from Freesurfer, SPM, and FSL, etc. A detailed quality control protocol will be used to ensure the image quality [50] and a radiologist at Karolinska University Hospital, Stockholm, Sweden, will assess the images.

*Saliva samples* Subjective stress of the participants before the saliva collection will be assessed with a Hospital anxiety depression scale questionnaire. Saliva will be collected to measure cortisol levels as a measure of stress. Stimulated saliva will be collected by having the participant chew on paraffin gum and spit out the saliva in a cup for 2 min. The amount of stimulated saliva will be noted and then approximately 5 ml will be collected in a tube and frozen at -80 degrees C for later analyzes. The time of conducting the test will be matched for all tests to avoid bias with fluctuating levels throughout the day. Participants will be instructed not to eat or drink for about two hours before the test.

*Ethical issues* The regional ethical review board in Stockholm has approved the study (Dnr: 2012/652-31/1). An amendment (Dnr 2016/670-31/2) on the updated protocol has been accepted by the ethical review board, Stockholm. All participants will be given oral and written information about the study, and a signed written informed consent will be obtained prior to randomization and initiation of any testing. The data generated from the study will be stored following the Swedish Archives Act and the Personal Data Act.

### Statistical methods

The data from the study will be entered in a Microsoft Excel sheet and exported to statistical software packages like SPSS Inc, Statistica, StatSoft Inc, or similar advanced analytics software packages for analysis. The result from the cognitive testing will be analyzed as repeated measure Analysis of covariance (ANCOVAs) with groups (experimental and control) and time (pre-and post-rehabilitation) as factors. Group-by-time interaction, the main effect of the group, and the main effect of time will be evaluated. To compare the magnitude of gains, the effect size (partial eta-square) will also be calculated. We will use time-length between pre-and post-tests as a covariate of interest with aiming to better control group differences. To predict the intervention outcomes, we will develop models that mainly predict cognitive test measures (NA). Predictive ability is defined as the amount of variance in the outcome that can be explained by pre-test cognitive status (slope method). Explained variance in the outcome will be obtained by analyzing linear regressions.

Preprocessing and statistical analyses of MRI data will be performed with Statistical Parametric Mapping (SPM) run in MATLAB (MathWorks). Movement correction will be performed by realigning and unwarp to the first image in the series. To consider group-specific anatomical brain differences, all patients will be normalized to Montreal Neurological Institute (MNI) echoplanar-imaging template. However, cortical thickness measures will not be normalized but rather used in their raw form. For cortical segmentation, a Freesurfer pipeline will be applied to the MRI images to produce regional cortical thickness and volumetric measures. To investigate rehabilitating-related changes repeated measures ANCOVAs will be performed with the groups (experimental and control) and time (pre-and post-rehabilitation) as factors. We will control for multiple comparisons with the Bonferroni-Holm method since all cognitive tests are correlated and because a majority of the test variables in this study belong to the same family (cognitive domain) or even the same test. In case of significant behavioral effects (i.e., a significant group-by-time interaction effect indicating that: the experimental group improves their cognitive performances > the control group), the changes will be evaluated as covariates of interest in relation to the potential brain changes.

## Discussion

We have, in the current protocol introduced an interventional study that aims to investigate the relationship between mastication and cognition in humans. It has previously been proposed that people who maintain their chewing ability may be less likely to develop dementia, compared to those who cannot chew well [13]. The decreased chewing function may be an unrecognized and potentially, modifiable risk factor contributing to the development of cognitive decline. Although an association between masticatory function and neurocognition seems evident from animal studies, interventional studies on humans are lacking, and a causal relationship has not yet been established. Therefore, during the course of the study, about eighty participants will be rehabilitated with modern contemporary prosthodontic treatment, and cognitive skills along with brain scans will be evaluated before and after the prosthodontic rehabilitation procedures. Additionally, the masticatory function will be assessed with subjective and objective measurements along with the overall oral health-related quality of life. If rehabilitation of masticatory function shows positive effects on neurocognitive performance, or prolonged cognitive maintenance (i.e., no further decline), it will have great implications on future health care for patients with impaired masticatory status at risk of developing dementia. Thus, the present project may provide a new avenue for the prevention of cognitive decline and dementia and bridge gaps between chewing function and cognition.

Our overall hypothesis that rehabilitation of the masticatory function will lead to improved cognitive performance primarily in the performance of episodic memory and learning measured by BVMT-R tests along with the corresponding neural changes mainly in the hippocampus and/or prefrontal cortex have substantial support from animal studies. In particular, Watanabe K, et al., in a series of experiments on aged SAMP8 mice showed that deliberate cutting the molar teeth results in impaired learning ability [51]. It was proposed that the probable mechanisms for this observation were that a molarless condition in aged mice decreases the *Fos* Induction in the hippocampus, which is related to impaired development of learning ability. Further, it was reported that the *Fos* induction in the hippocampus was normalized by the restoration of the molarless mice with artificial crowns. The mice also demonstrated better performance in the water maze test after the replacement with artificial teeth than during the molarless condition. In other words, restoring masticatory function with artificial crowns in aged mice counteracted the reduction in spatial memory and hippocampal neuron function [51]. Our study focuses on frontotemporal mediated cognitive functions such as episodic memory and executive functions which have support from studies on cognitive neuroscience. A robust association has been found between cognition (mainly episodic memory, and executive function) and self-reported chewing ability. It was suggested that chewing difficulty was more likely to be associated with cognitive impairment irrespective of whether the participants chewed with natural teeth or with a dental prosthesis [52]. Paganini-Hill and colleagues have also recognized and emphasized the importance of teeth and dental rehabilitation with prostheses (removable or fixed) to support mastication and reduce the risk of cognitive decline and development of dementia [53]. Accordingly, our MRI protocol can capture potential brain tissue changes with the structural sequences, and functional changes using a combination of resting-state BOLD fMRI and brain perfusion sequences, as well as our cognitive test battery with its research-based focus on episodic memory and executive functions.

The study follows a clinical, longitudinal randomized trial design and has considered any possible bias due to type 1 error. Accordingly, the two pre-tests in the control group are included to rule out for test–retest effects of the cognitive measurements. The likelihood of the participants performing better in the cognitive tests in the second assessment is quite high. Therefore, the post-test in the control group will rule out any type 1 error due to an improvement over time that could potentially be interpreted as an improvement due to the intervention (dental rehabilitation). We hypothesize that at post-test (after the intervention), the experimental group will perform better in cognitive tests than the control group at post-test (i.e., the intervention improves the cognitive abilities better than what the test–retest effect does). However, at post-test 2 (after the intervention), we hypothesize that the control group will perform at a similar level as the experimental group as a result of gained effects due to the intervention. Importantly, the control group will probably have a shorter time length between pre-and post-test than the experimental group. This is a limitation that might lead to a greater risk of type- 2 errors and thereby decrease the possibilities to detect intervention effects. However, due to ethical reasons, we cannot deny the control group their dental rehabilitation any longer than 3–4 months after the pre-tests (see the method section for further information on the control for time differences between pre-and post-test). In contrast, if we detect positive intervention effects, we will be able to draw firm conclusions (i.e., despite a shorter time between pre-and post-test for the control group). Importantly, we will also evaluate if we can identify pre-intervention predictors for intervention outcomes. This is especially important regarding cognitive status (assessed with the pre-intervention neuropsychological tests) and we will therefore statistically model cognitive performances to predict intervention outcomes.

The (optional) MRI acquisitions are in the protocol design not conducted on all study participants, even when taking medical contraindications and reluctance to participate in consideration. The reason to include 40 participants (20 in each group) is to a large extent dependent on the feasibility and availability of the resources. Moreover, this could be seen as a weakness in this part of the study since it will be more difficult to achieve strong reliability in collected data.

To optimize the cooperation, adaption, and survival of the intervention the participants will be rehabilitated with a fixed tooth or/and implant-supported prosthesis, also including overdentures (OD, implant, or tooth-supported prosthesis). Studies conducted comparing the masticatory function with different prosthetic treatments have shown a better function with fixed alternatives compared with a partial or full removable denture [54]. Although overdentures are considered as removable appliances they are included since they improve the masticatory performance compared to full removable ones that are the alternative treatment, especially when used in the mandibular [55]. OD is often the preferable option when having anatomical and/or economical limitations and by including OD it will broaden the socioeconomic span as well as the possibility to retain more participants to post-tests. Previous studies have reported a higher survival and lower complication rate in patients rehabilitated with the fixed prosthesis in comparison to the removable ones. In particular, authors have reported a survival rate of 98.7 [56] and 95.4% [57] for fixed implant-supported prostheses over 5 years. In a retrospective study on fixed tooth-supported partial dentures over 5 units; conducted partly at the same department as the current study, the 10-year survival rate was 74.4% [58]. In order to have better treatment outcomes and eliminate any confounders related to the treatment procedures, the participants in the current study will only be treated by specialized dentists or dentists in specialization training under supervision. Though, we do acknowledge that there are a few limitations of the current study protocol. Confounding factors such as sarcopenia, adaptive capacities and adjustments of diet consistency due to loss of teeth can influence the secondary outcome parameters. However, a recent preliminary study with similar study objectives has with a smaller sample shown promising results [59]. Successive studies will be planned to consider all the major confounding factors reflecting on the results of the current study and perhaps follow the participants in the current study for a longer duration.

Contemporary orofacial neuroscience [60] related research has emphasized the importance of sensorimotor regulation [61–70] and adaptation to the altered oral environment during biting [64, 71–74] and chewing behaviors [71]. Further, recent studies have discussed the significance of retaining natural dentition and optimizing oral functions in people with a dental prosthesis [62, 63, 75–78]. These studies have in general suggested that restoration of chewing function can be an important factor for healthy wellbeing, for review see, [79, 80]. If rehabilitation of the masticatory function also shows positive effects on neurocognitive functions, this will have great implications for future health care of patients with impaired masticatory status. Thus, the present project may provide a new avenue for the prevention of cognitive decline and dementia.

## Data Availability

The datasets generated and/or analyzed during the current study are not publicly available but are available from the corresponding author on reasonable request.

## Abbreviations

ANCOVAs: Analysis of Covariance
AUDIT: Alcohol Use Disorders Identification Test
BDNF: Brain-Derived Neurotrophic Factor
BOLD: Blood Oxygen Level-dependent
BVMT-R: Brief Visuospatial Memory Test-Revised
D-KEFS: Delis-Kaplan Executive Function System
HAD: Hospital anxiety and depression
MMSE: Mini-Mental State Examination
MNI: Montreal Neurological Institute
MRI: Magnetic Resonance Imaging
NA: Neuropsychological Assessments
NVS: Department of Neurobiology, Care Sciences, and Society
OHIP: Oral Health Impact Profile
OD: Overdentures
pCASL: Pseudo-Continuous Arterial Spin Labeling
PDHS: Public Dental Health Service
RAVLT: The Ray Auditory Verbal Learning Test
SAMP: Senescence Accelerated Mouse Prone
SPM: Statistical Parametric Mapping
SUBIC: Stockholm University Brain Imaging Centre
TMT: Trail Making Test
VF: Verbal Fluency
VOH: Variance of Hue
WAIS: Wechsler Adult Intelligence Scale
